# Tool Wear Prediction in Machining of Aluminum Matrix Composites with the Use of Machine Learning Models

**DOI:** 10.3390/ma17235783

**Published:** 2024-11-25

**Authors:** Adam Hamrol, Maciej Tabaszewski, Agnieszka Kujawińska, Jakub Czyżycki

**Affiliations:** Faculty of Mechanical Engineering, Poznan University of Technology, 60-965 Poznań, Poland; adam.hamrol@put.poznan.pl (A.H.); maciej.tabaszewski@put.poznan.pl (M.T.); jakub.czyzycki@put.poznan.pl (J.C.)

**Keywords:** tool wear prediction, aluminum matrix composite, diagnostic model, machine learning

## Abstract

This paper discusses the diagnostic models of tool wear during face milling of Aluminum Matrix Composite (AMC), classified as a difficult-to-cut material. Prediction and classification models were considered. The models were based on one-dimensional simple regression or on multidimensional regression trees, random forest, nearest neighbor and multilayer perceptron neural networks. Measures of diagnostic signals obtained from measurements of cutting forces and vibration accelerations of the workpiece were used. The study demonstrated that multidimensional models outperformed one-dimensional models in terms of prediction accuracy and classification performance. Specifically, multidimensional predictive models exhibited lower maximum and average absolute prediction errors (0.036 mm vs. 0.050 mm and 0.026 mm vs. 0.045 mm, respectively), and classification models recorded fewer Type I and Type II errors. Despite the increased complexity, the higher predictive accuracy (up to 0.97) achieved with multidimensional models was shown to be suitable for industrial applications. However, simpler one-dimensional models offered the ad-vantage of greater reliability in signal acquisition and processing. It was also highlighted that the advantage of simple models from a practical point of view is the reduced complexity and consequent greater reliability of the system for acquiring and processing diagnostic signals.

## 1. Introduction

Meeting requirements for dimensional and shape accuracy and the properties of the surface and surface layer of machined parts is the main goal of any machining process [1,2]. One of the factors that determine machining quality is the cutting tool, including its condition as determined by the degree of wear of the cutting edge or cutting edges (in multi-tools). To achieve the required machining quality, tool life, i.e., the time after which it must be replaced or regenerated, is planned conservatively to ensure that quality requirements are met with the greatest possible certainty. To define this safe tool life, various statistical and analytical methods are used [3,4,5,6].

In order to rationally use the cutting potential of a tool, it is possible to monitor the machining results, i.e., to evaluate the characteristics that determine the quality requirements, by measuring them offline, online or inline [7]. The possibilities for online or inline measurement of machining results are increasing, mainly due to vision systems. Vision systems are also used to assess the condition of cutting tools [8]. However, they are still costly and often inaccurate and unreliable. Therefore, the diagnostics of the machining process based on cutting force signals or vibroacoustic signals, especially vibration ones, still dominates and is currently being developed [9,10].

The value and variation of the cutting force depends on the cutting parameters (thickness and width of the layer being cut, cutting speed), the geometry of the cutting edge, and the conditions at the interface between the cutting edge surface and the workpiece materials [11]. As the tool flank wears, its contact surface with the workpiece increases, which causes an increase in cutting force. The cutting force is also influenced by an increase in the radius of the corner rounding and the wear on the rake face. The groove that forms on this surface causes an increase in the effective rake angle, which promotes a decrease in cutting force.

Because the cutting force varies dynamically, it causes varying deformations of the M-F-W-T system (Machine tool-Fixture-Workpiece-Tool). The movements of the machine tool components, in particular the rotation of the tool (in milling) or of the workpiece (in turning), are transformed into vibro-acoustic processes, vibrations, and acoustic emissions that occur in and around the machine tool’s mechano-acoustic system. The externally measured signal is the response of the dynamic system of the machining system with a certain transmission to the sum of the cutting force excitations [12,13].

There are many examples in the literature of the use of cutting force signals and vibroacoustic phenomena for cutting tool condition diagnosis. During machining, diagnostic signals and the corresponding degree of cutting edge wear is recorded. Tool wear is most often determined by direct geometric indices, measured mainly on the contact surface and sometimes on the face surface of the cutting edge. Based on the recorded signals, specific signal measures are determined, which are then used to build the corresponding diagnostic model [14,15,16,17].

When designing a diagnostic system, special attention should be paid to the selection of signal measures directly or indirectly related to the wear of the cutting edge. The recorded signals contain much more information that is not covariant with the condition of the blade and is unnecessary from the point of view of making diagnostic decisions—e.g., related to the machine drive.

The ultimate goal should be to select such measures that provide a reliable assessment of the wear of the cutting edge. Here, machine learning methods can be helpful, which will allow you to select only those related to the condition of the blade (e.g., decision trees) from the redundant set of measures [18,19].

The process of blade wear itself can be visible in the vibration signal in at least two aspects. First, a worn tool will be a source of stronger dynamic interactions during the contact of the tool with the material, which can manifest itself in specific frequencies related to the kinematics of the cutting process, as well as in the natural frequencies of the system. Additionally, changes in a wide band of frequencies related to the phenomenon of friction that increases with tool wear can be expected [20].

Many algorithms are used for this purpose, starting with all kinds of methods for creating combinations of primary measures and selecting the most informative ones [17,18,19,20]. Examples are as follows: PCA—Principal Component Analysis, KPCA—Kernel Principal Component Analysis, LDA—Linear Discriminant Analysis, ICA—Independent Component Analysis [21], SVD—Singular Value Decomposition [22,23], RFE—Recursive Feature Elimination, FD—Fisher’s Discrimination, and CA—Correlation Analysis [24,25].

Two basic methods of diagnostic inference, prediction and classification, are used to assess the degree of tool cutting edge wear. Predictive methods make it possible to predict the degree of tool wear, expressed for example by the amount of wear. In the classification method, the assessment of the tool condition is expressed descriptively (with a label), in the simplest case in a binary form. Two-state identification is a practical approach under industrial conditions [26,27,28].

The suitability or unsuitability of a tool must be predetermined by an indicator of wear, such as edge wear, surface deterioration and others. In turn, exceeding certain diagnostic measures that best assess the condition of the cutting edge will allow classification. For this purpose, in addition to performing a series of tests, the experience of the operator or technologist is also needed. In practice, it is not obvious to define these two states, and CNC machine operators are often guided by intuition and the decision to replace the tool is not always the right one [29].

Inference methods, both predictive and classification-based, are based on various mathematical models. The parameters of the model constituting the basis for diagnostic inference can be determined on the basis of data obtained in specially conducted experiments or on the basis of data obtained in real machining conditions, using learning algorithms, incl. machine learning. For the purposes of machine learning and diagnostic inference, many different mathematical models are offered in the literature [30,31,32].

The tool wear prediction model should be selected according to the type of cutting edge wear. For example, if the wear process is dominated by surface abrasion, the use of a simple linear or nonlinear regression model may be sufficient. However, if several forms of wear occur simultaneously, the use of multidimensional models is required. Such a multidimensional wear mechanism occurs, e.g., when cutting Metal Matrix Composites (MMCs). These composites are used in modern automotive, aerospace and marine industries due to their unique mechanical properties. Parts made from this material are lighter than those made from steel, while maintaining similar strength properties, rigidity and the ability to operate at high temperatures and under hard operating conditions.

An innovative example of the use of metallic composite materials is the Hubble tele-scope’s 3.6 m long antenna boom offering the desired stiffness. It is made of an Al6061 alloy matrix composite reinforced with graphite fibers. Another example of the use of MMCs is the Space Shuttle Orbiter. For its construction, B/Al composite was used for parts of the fuselage and landing gear, saving 45% of the weight [33].

Metal Ceramic Composites (MCCs) are composed of at least two phases clearly separated by an interfacial boundary. One of these phases is a reinforcing phase (SiC or Al_2_O_3_). The metal matrix is formed by light metal alloys (Al, Ti, Mg). Depending on the type of structural material, the reinforcement typically accounts for 10–40% of the composite composition. There are two basic methods for producing MCCs: ex situ, in which the reinforcing phase is prepared in a separate process and is separately introduced into the matrix, comprising composites reinforced with SiC particles, and in situ, where a reinforcing phase is formed during the metallurgical process as a result of chemical reactions [34].

**Figure 1:**
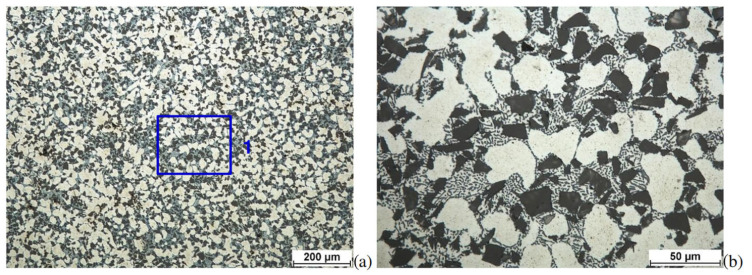
Microstructure of the Aluminum Ceramic Composite (AMC) used in the study (DURALCAN F3S.10S). The microstructures of the samples were revealed after etching in the Mi2Al reagent according to the PN-75/H-04512. The microstructure observations of the above samples were performed at image magnifications of 100× (**a**) and 500× (**b**) [35].

An example is the Aluminum Ceramic Composite (AMC) used in the study. The microstructure of such a composite is shown in Figure 1.

Due to the addition of hard silicon carbide SiC particles in the structure, these materials are classified as hard to cut [36]. Research shows that the significant size of SiC particles and their percentage increase in the mass of the workpiece leads to accelerated tool wear [37]. This is due to the more frequent contact of the cutting edges and corners with the hard carbides. The result of the above phenomenon is the occurrence of adhesive and abrasive wear during machining, which have a destructive effect on the tool structure [38,39]. They are manifested by the formation of a build-up which, under the influence of the forces applied, can detach from the cutting edge elements or adversely affect tool wear and the associated increase in temperature during operation.

The aim of the work is to evaluate various prediction and classification models determined by machine learning methods in diagnosing the condition of cutting tools during Ceramic Composite milling characterized by 10% of reinforcement in the form of ceramic particles.

The research results fill two research gaps. The first concerns the identification of the wear pattern of the cutting edge during milling of the Aluminum Ceramic Composite. The second gap concerns the comparison and assessment—using the example of the considered machining case—of the usefulness of different diagnostic inference models.

## 2. Materials and Methods

### 2.1. Processing Conditions

**Figure 2:**
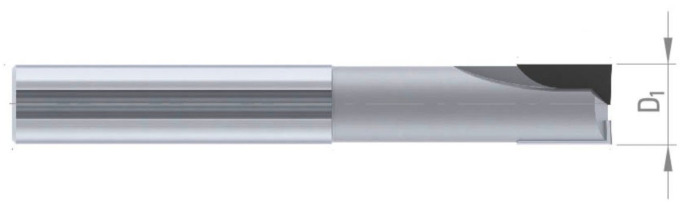
PCD end mill cutter (diameter D_1_ = 10 mm).

A monolithic cylindrical end mill cutter with polycrystalline-type diamond (PCD) cutting edges was used (Figure 2) for machining. Tools were mounted in thermal holders. Machining was carried out with the parameters listed in Table 1. The parameters used are typical for fine and extra-fine machining. The tests were carried out on a DMC70V machining center with a maximum spindle speed of *n* = 30,000 rpm.

The blade wear value VBc was calculated as the average of the edge wear measurements of both blades. The blade edge wear was measured using a ZEISS Discovery V20 stereo laboratory microscope.

### 2.2. Measurement of Cutting Force and Vibration Acceleration

During the milling operation, the cutting force components and the acceleration of vibration were measured in the following directions: feed direction (X), normal feed direction (Y) and axial direction (Z).

**Figure 3:**
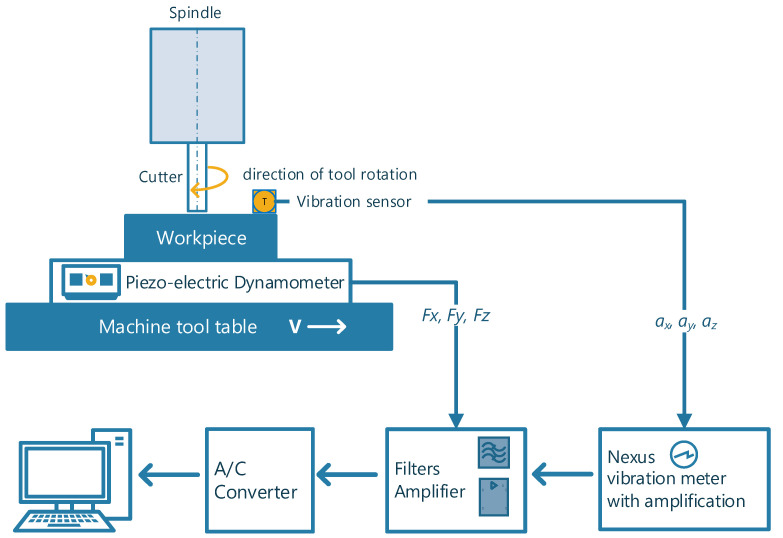
Scheme of the force and vibration measurement system.

To measure vibration acceleration, a three-component piezoelectric sensor from Brüel & Kjær mounted on a thread on the workpiece was used, from which signals were transmitted to a NEXUS Brüel & Kjær amplifier. The components of the total force were measured using a piezoelectric platform from which signals were transmitted to KISTLER load amplifiers. The diagram of the measurement system is shown in Figure 3.

### 2.3. Creation of Signal Measures

Force and vibration acceleration signals were recorded during successive machining operations. A total of 140 signals from process realizations with a cutting speed of *v_c_* = 500 m/min and 159 signals from a cutting process with a speed of *v_c_* = 900 m/min were recorded. After a certain number of end mill passes, the flank wear VBc of the tool was measured.

**Figure 4:**
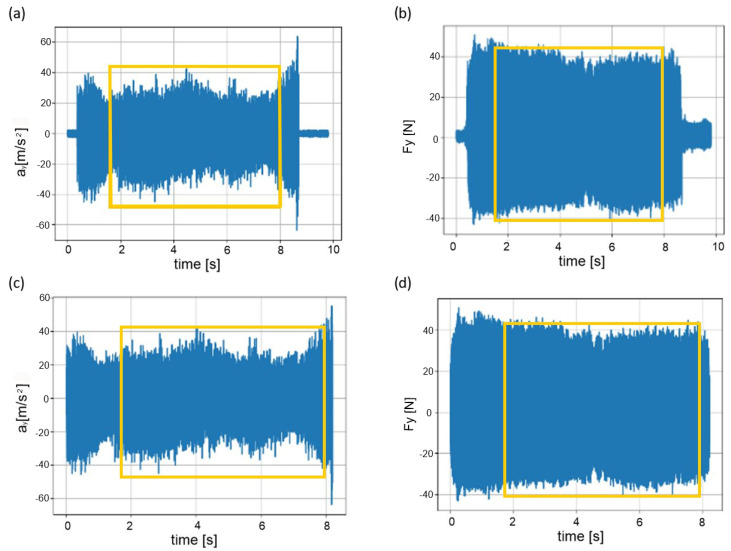
(**a**–**d**). Example signal recordings of vibration acceleration and force (**a**,**b**), and cut sections of tool input and output in and out of the workpiece material (**c**,**d**).

Signal segments corresponding to the full penetration of the cutting edge into the workpiece material were analyzed (periods of entry and exit of the cutting edge from the material were omitted). Example recordings of the vibration acceleration signal (in the Y—*a_y_* direction) and the force signal (in the Y—*F_y_* direction), complete and after removal of the signal fragment unrelated to the cutting process, are presented in Figure 4. In addition, as indicated in Figure 4, the input and output phases of the cutting edge from the material were discarded.

Frequency bands of 500 Hz detected by the algorithm as active were combined into wider bands if they were adjacent. In these bands, the rms value of acceleration signals was determined. Using the results of the spectral analysis across the band, the Rice frequency was also calculated.

Other measures were determined in the time domain over the full measurement bandwidth. The following parameters calculated from one-second fragments of recordings were used as measures of force and vibration signals: rms values, the peak value of the signal, the average peak value (five averages divided into time intervals of 0.2 s), amplitude, average value, clearance factor, form factor, crest factor, impulse factor, kurtosis and Rice frequency.

A measure of the average peak value calculated from several maximum values was used to reduce the importance of random (unique) impulse phenomena that may not be related to tool wear. Finally, sixty-one signal measures were determined. In addition, the cutting speed *v_c_* was also used as one of the parameters for model learning.

### 2.4. Creating Data for the Training Set and the Test Set

Assuming that the defined signal measures (SMs) could be correlated with each other, an analysis of the relations between the individual SMs was performed, and all relations strongly correlated with others and simultaneously weakly correlated with VBc were initially rejected. Finally, thirty-four signal measures were included in further analysis (Table 2. Cutting speed *v_c_* was used as the distinguished independent quantity.

Table 2 presents symbols of acceleration signal measures (symbol *a*) and forces (symbol *F*). The indices *x*, *y* and *z* indicate the direction of measurement. Some of the measures were determined in selected frequency bands, as indicated in the last column. The definitions of individual measures can be found, for example, in [40].

A total of 1456 structured data records were created (SM_1_, SM_2_, … SM_34_; *v_c_* and VBc). With the aim of using signal measures to train diagnostic models, all records were divided into two sets:

- Training set (75% of the records);
- Test set (25% of the records); records were assigned to the subsets in a random manner (non-return draw).

### 2.5. Diagnostic Models

Predictive and classification diagnostic models were tested. A predictive model means that the output of the model is a predicted value of a specific form of cutting edge wear. In the case of classification model, a cutting edge is classified into a specific category based on its suitability for processing. Binary classification was used, i.e., the cutting edge wear is assessed as acceptable or unacceptable.

All diagnostic models were built using supervised machine learning. Due to the number of input signals and the method of inference, the following models were considered [41]:

(a) One-dimensional.
  - Simple regression (SR).
(b) Multidimensional.
  - Multiple linear regression (MLR);
  - Elastic net regression (ENR);
  - Classification and regression trees (CART);
  - Random forest (RF);
  - Nearest neighbor algorithm (NNA);
  - Multilayer perceptron (MLP).

### 2.6. Quality Measures of the Diagnostic Models

To assess the quality and effectiveness of the obtained models, the following measures were used:

(a) With regard to the predictive models:
  - Root Mean Square Error (RMSE);
  - Standard Deviation of RMSE (σ_RMSE);
  - Coefficient of Determination (R^2^).
(b) with regard to the classification models the measure listed below in the so-called confusion matrix (Table 3).

Where:

- True Positives (TP): when the actual value is Positive class and prediction is also Positive.
- True Negatives (TN): when the actual value is Negative class and prediction is also Negative.
- False Positives (FP): when the actual is Negative, but the prediction is Positive. Also known as the Type I error.
- False Negatives (FN): when the actual is Positive, but the prediction is Negative. Also known as the Type II error.

Based on the values defined in Table 3, the following measures were calculated:

- Accuracy: A measure of how often the classifier makes the correct prediction. It is the ratio between the number of correct predictions and the total number of predictions:
  Accuracy = (TP + TN)/(TP + TN + FN + FP)(1)
- Precision: A measure of correctness that is achieved in true prediction. It determines how many predictions are actually positive out of all the total positive predicted:
  Precision = TP/(TP + FP)(2)
- Sensitivity: A measure of actual observations which are predicted correctly, i.e., how many observations of positive classes are actually predicted as positive. It is defined as the ratio of the total number of correctly classified positive classes divide by the total number of positive classes:
  Sensitivity = TP/(TP + FN)(3)
- F1: A number between 0 and 1 and the harmonic mean of precision and sensitivity:
  F1 = 2 · TP/(2TP + FN + FP)(4)

## 3. Results

### 3.1. Tool Wear

**Figure 5:**
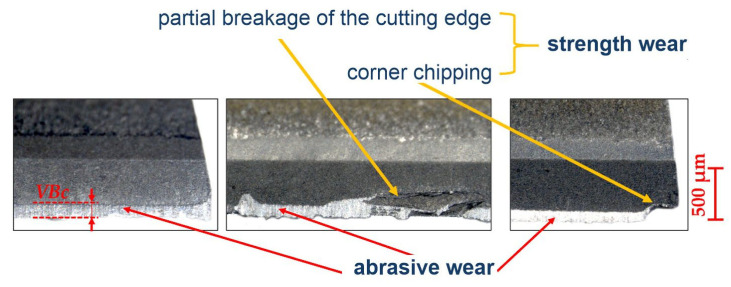
Photographs of tool wear and measuring method for the cutting edge wear.

The photos in Figure 5 show examples of tool wear observed during the tests. In each of the cases shown, the wear in the form of abrasive wear of the flank surface dominates. There is also strength wear on the edge of the blade, in the form of chipping of the corners and partial fracture of the cutting edge. However, these forms of wear are random, i.e., they appear only in some cases. Based on the above, the width of abrasive wear of the flank surface, VBc [mm], was adopted as the tool wear criterion.

**Figure 6:**
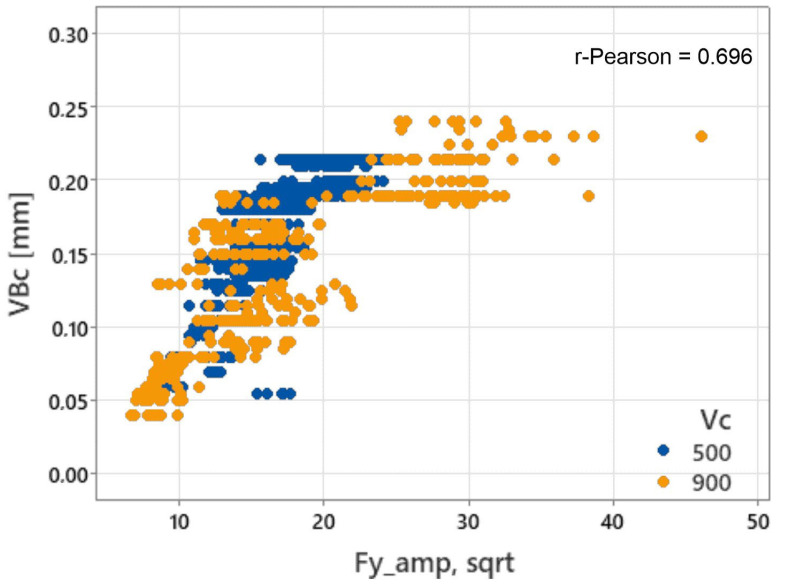
Example of the relationship between the VBc value and the signal measure *Fy_amp_sqrt* in the training set.

To illustrate the nature of the relationship between the tool wear and the diagnostic measures, Figure 6 shows a sample relationship for one of the signal measures (*Fy_amp_sqrt*). A significant scatter of values can be seen, which is reflected in the relatively low Pearson correlation coefficient (0.696). The graph also shows that the measured values obtained during milling at a speed of *v_c_* = 900 m/min “overlap” with the values obtained during cutting at a speed of *v_c_* = 500 m/min.

**Figure 7:**
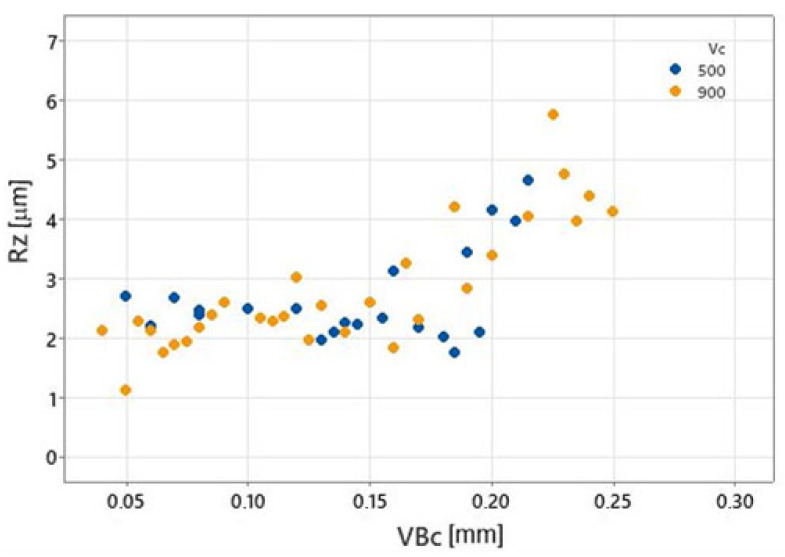
Relationship between VBc and the surface roughness Rz.

In order to determine the critical value VBc for the classification model, the relationship between VBc and the surface roughness parameter Rz was determined (Figure 7).

It can be seen that from the value of VBc = 0.2 mm the roughness Rz shows an intensive increase. Assuming that the value Rz is critical for the quality of the processing, and its permissible value is 4 μm, VBc = 0.2 mm was fixed as the limit value of wear in the binary classification.

### 3.2. Predictive Diagnostic Models

#### 3.2.1. One-Dimensional

To determine the predictive diagnostic model based on the simple regression out of all the signal measures (Table 2), four measures, *Fy_avg, Fy_rms, Fy_mean peak* and *Fy_amp,sqrt*, the most strongly correlated (high R^2^ in Table 4) with the variable VBc, were chosen. The quality measures of the obtained models are presented in Table 4.

**Figure 8:**
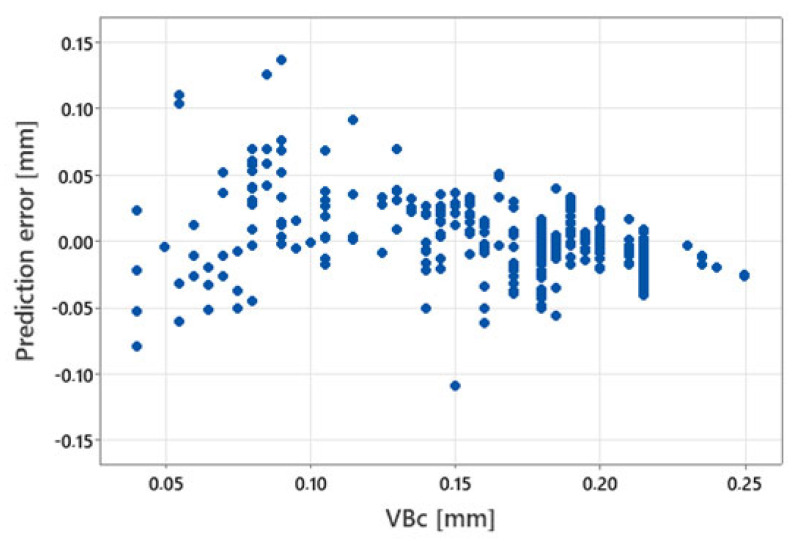
Prediction error for predictive diagnostic models based on simple regression.

The best results were obtained for the measure Fy_amp, sqrt. The plot of the prediction error for this model is shown in Figure 8.

#### 3.2.2. Multidimensional

Four multidimensional predictive diagnostic models based on MLP, ENR and CART were created. The models’ parameters were selected using k-fold cross-validation for *k* = 10. In the case of the SR model and the ENR method, the suitability of individual SM_i_ was also assessed using recursive feature elimination with cross-validation [42]. Other modifiable hyperparameters fitted during model building were as follows:

- For the ENR, the proportion of two sets regularizations and the multiplicative factor of the corresponding expressions in the penalty function.
- In regression using the CART, the maximum tree depth.
- For the RF, the maximum number of MS_i_ considered at a given split, the number of trees in the forest and the inclusion or not of bootstrap samples [33].

Due to the set of measurement signals, two variants were tested:

(a) Full set of signal measures;
(b) Reduced set of signal measures.

(a) Full set of signal measures

Table 5 shows the quality measures obtained for each model.

**Figure 9:**
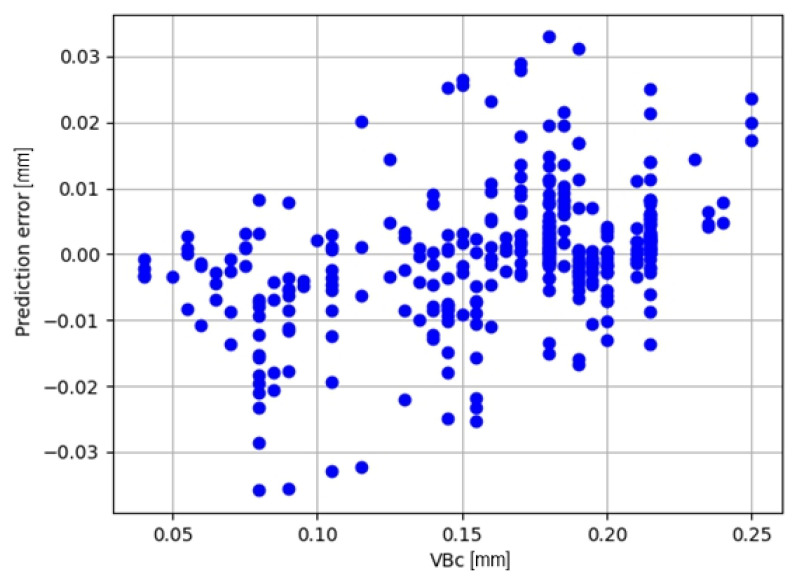
Prediction error for the RF model.

The best model obtained during the cross-validation test, model RF, achieved a coefficient of determination on the test set of R^2^ = 0.9573. The prediction error for this model is shown in Figure 9.

(b) Reduced set of signal measures

By analyzing the relevance of MS_i_ in both the CART and RF methods, it was found that many MS_i_ are not relevant or are highly insignificant. These include information on the cutting speed *v_c_* and SM based on vibration accelerations in the y-direction (designation *a_y_*). Therefore, it was decided to attempt to simplify the system by eliminating some MS_i_ associated with specific measurement directions or physical quantities. In particular, the removal of the parameter *v_c_*, which has to be entered into the system by the operator and can therefore be prone to errors, seems significant.

**Figure 10:**
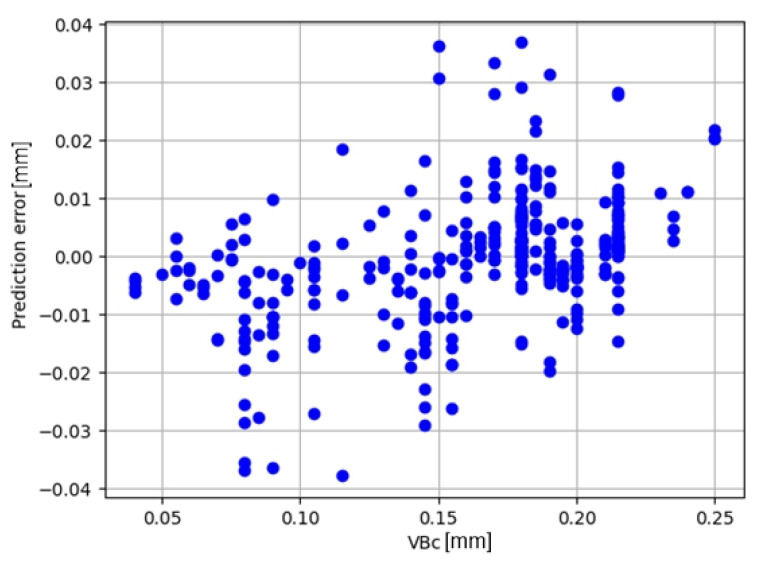
Prediction error for the simplified RF model.

The value of the coefficient of determination determined on the test set for the best RF model decreased from R^2^ = 0.957 to R^2^ = 0.949 (RMSE = 0.1896, σ-RMSE = 0.0021), while the maximum absolute prediction error did not exceed 0.038 mm. The model still seems useful. The resulting prediction errors for the test set are shown in Figure 10.

### 3.3. Classification Models Derived from Prediction Models

Three classification models derived from prediction models were tested:

(a) Simple regression (SR).
(b) Random forest (RF) with the full set of signal measures.
(c) Random forest (RF) with the selected set of signal measures.

The obtained model quality measures are presented in Table 6, Table 7 and Table 8.

### 3.4. Classification Models Constructed Directly from Raw Data

The following classification models constructed directly from raw data were tested: CART, RF, NNA and MLP. Two cases were analyzed:

(a) Full set of signal measures;
(b) Reduced set of signal measures.

From the considered models, the best model was initially selected based on the cross-validation test performed on the training set. The test results are presented in Table 9.

The highest quality for the full set of SMs was demonstrated by the MLP model. Its quality parameters on the test set are presented in Table 10. For the reduced set of SM_i_, the best model was RF. Results are presented in Table 11.

## 4. Discussion of the Results

During machining of the Aluminum Matrix Composite materials (AMC), the dominant form of wear of the cutting edge is abrasive wear of the flank surface. However, there are also—sporadically—forms of strength wear (Figure 5), e.g., chipping of the cutting edge. As a result, the relationship between the amount of cutting edge wear and diagnostic signal measures is relatively weak statistically (compare Figure 5 and Figure 6). Only in some cases, the relationship between cutting edge wear and a single signal measure may be strong enough to effectively assess the condition of the cutting edge on its basis.

However, in general, multidimensional models should be considered to have an advantage over simple models in terms of diagnostic performance. This is confirmed by the quality measures of the diagnostic models created (Table 12). They allow for comparison of the accuracy of all types of models considered in this paper. The table presents only the results of the best variants in the individual model groups discussed.

The presented results clearly indicate that the advantage of multidimensional models applies to both prediction and classification models, and is reflected in quality measures as accuracy, precision and sensitivity.

From the tables in Section 3.2 and Section 3.3, it can also be concluded in particular that the multidimensional predictive models have a smaller maximum prediction error and average absolute prediction error than the one-dimensional model (0.036 mm vs. 0.050 mm and 0.026 mm vs. 0.045 mm, respectively). Multidimensional classification models also show a lower number of Type I and II errors.

It can be stated that the accuracy at the level of 0.7 that was achieved for the considered machining case is a very good result in industrial conditions. As wear approaches the accepted wear limit (0.2 mm), the level of expected error allows reasonable diagnostic decisions to be made. It does however indicate that the models tend to ignore the exceedance of the wear limit value. However, the percentage of these cases seems to be small.

The sensitivity of the model to possible interference from measurement systems and the supervised process must also be considered. In the case of multidimensional models that operate on multiple measures of measurement signals, there is a risk that disturbed data, both at the learning and testing stages of the model, and subsequently during its application, can significantly affect the generated results. Distortions can be the result of failure or calibration of the measurement system, e.g., sensors, acquisition paths and signal processing. To recognize these distortions, it is necessary to use systems that monitor the correct operation of the measurement system. Disturbances may also originate from anomalies associated with atypical conditions of the supervised process or specific forms of tool wear.

Therefore, in specific situations, the choice of inference model should include con-sideration of adopting a trade-off between the precision of the prediction or classification and the reliability of the data fed into the model.

In this paper, such a possibility was proven on the example of a regression RF model. It was shown that it is possible to obtain an efficient classification model even if the measurement system is significantly simplified and after eliminating the parameter *v_c_*.

As already emphasized, the obtained results concern the machining of a specific composite. Since there are no studies in the literature on the diagnostics of the milling process of this composite, it is not possible to compare the obtained results with the results obtained by other researchers. However, the findings regarding the advantage of multidimensional models over one-dimensional models are convergent with other researchers [41,42,43,44].

## 5. Conclusions

The article presents the usability of different diagnostic models to assess tool wear during face milling of Aluminum Matrix Composite (AMC). Prediction and classification models were created and tested. Random forest, nearest neighbor, and multilayer perception neural networks were used for inference about the edge wear, one-dimensional simple regression and multidimensional regression trees. As the input, values for the diagnostic models’ various measures of signals obtained from measurements of cutting forces and vibration accelerations of the workpiece were used.

Tests showed that multidimensional models are generally more effective than one-dimensional models in both classification and prediction tasks. Tests allowed prediction and recognition of the tool wear with an accuracy at the level 0.97, which is practically accepted.

Although the one-dimensional model is less accurate, its advantage is the lower level of complexity and the resulting greater reliability of the system for acquiring and processing diagnostic signals.

Future research should demonstrate the extent to which the conclusions identified in the research presented in this article can be transferred to other processing conditions. The tests will be extended to other types of composites and their machining using various cutting speeds and other machining parameters.

## Figures and Tables

**Table 1:** Tool and machining parameters.

Tool Parameters	Value	
end mill cutter diameter D1 [mm]	10	
number of cutting edges z	2	
Machining parameters	1st set	2nd set
spindle speed n [rpm]	15,923	28,662
cutting speed vc [m/min]	500	900
cutting feed rate vf [mm/min]	1115	2006
feed per tooth fz [mm/edge]	0.035	0.035
axial cutting depth ap [mm]	5	5
radial depth of cut ae [mm]	0.1	0.1

**Table 2:** Measures taken into account in training diagnostic models.

	Symbol	Description	Frequency Band
1	ax_rms_(1000.0, 2500.0)	Rms value of vibration acceleration	1.0–2.5 kHz
2	ax_rms_(7000.0, 8000.0)	Rms value of vibration acceleration	7.0–8.0 kHz
3	ax_mean_peak	Average peak value of vibration accelerations	Full
4	ax_CF	Crest factor of the vibration acceleration signal	
5	ax_FF	Form factor of the vibration acceleration signal	
6	ax_f.Ricea	Rice frequency	
7	ay_rms_(1500.0, 2500.0)	Rms value of vibration acceleration	1.5–2.5 kHz
8	ay_rms_(3000.0, 3500.0)	Rms value of vibration acceleration	3.0–3.5 kHz
9	ay_rms_(7500.0, 8000.0)	Rms value of vibration acceleration	7.5–8.0 kHz
10	ay_rms_(11,000.0, 11,500.0)	Rms value of vibration acceleration	11.0–11.5 kHz
11	ay_mean_peak	Average peak value of vibration accelerations	Full
12	ay_avg	Average value of the vibration acceleration signal	
13	ay_CF	Crest factor of the vibration acceleration signal	
14	ay_FF	Form factor of the vibration acceleration signal	
15	ay_IF	Impulse factor for vibration acceleration	
16	ay_kurt	Vibration acceleration kurtosis	
17	az_mean_peak	Average peak value of vibration accelerations	Full
18	az_avg	Average value of the vibration acceleration signal	
19	az_CF	Crest factor of the vibration acceleration signal	
20	az_FF	Form factor of the vibration acceleration signal	
21	az_IF	Impulse factor for vibration acceleration	
22	az_kurt,	Vibration acceleration kurtosis	
23	az_f.Ricea	Rice frequency	
24	Fy_rms	Effective value of the dynamic component of the force	Full
25	Fy_mean_peak	Average peak force	
26	Fy_avg	Average value of the force signal	
27	Fy_amp_sqrt	The root amplitude of the force signal	
28	Fy_CF	Crest factor of the force signal	
29	Fy_FF	Form factor of the force signal	
30	Fz_rms	Effective value of the dynamic component of the force	Full
31	Fz_mean_peak	Average peak force	
32	Fz_avg	Average value of the force signal	
33	Fz_CF	Crest factor of the force signal	
34	Fz_FF	Form factor of the force signal	
35	v_c_	Cutting speed	-

**Table 3:** Confusion matrix for binary problem [42].

	Predicted Class Positive (P)	Predicted Class Negative (N)
Real class: Positive +	True Positive (TP)	False Negative (FN)
Real class: Negative −	False Positive (FP)	True Negative (TN)

**Table 4:** Quality measures of the predictive one-dimensional diagnostic model (simple regression).

Measure MS	RMSE	σ_RMSE	R^2^
Fy_avg	0.063780	0.0270937	0.522
Fy_rms	0.067685	0.0263348	0.695
Fy_mean peak	0.069352	0.0302427	0.549
Fy_amp, sqrt	0.0490801	0.0270609	0.696

**Table 5:** Quality measures of the predictive multidimensional diagnostic models (full set of signal measures).

Model	RMSE	σ-RMSE	R^2^
SR	0.0221	0.0020	0.7543
ENR	0.0241	0.0028	0.7037
CART	0.0179	0.0015	0.8605
RF	0.0117	0.0009	0.9573

**Table 6:** Quality measures of the classification diagnostic model based on SR prediction model (Fy_amp_sqrt measure).

	Forecast Class VBc ≥ 0.2 mm	Forecast Class VBc < 0.2 mm
Actual class VBc ≥ 0.2 mm	72 (TP)	23 (FN)
Actual class VBc < 0.2 mm	26 (FP)	243 (TN)
Accuracy	0.865	
Precision	0.735	
Sensitivity	0.758	
F1	0.746	

**Table 7:** Quality measures of the classification diagnostic model based on the RF multidimensional prediction model (full set of SM_i_).

	Forecast Class VBc ≥ 0.2 mm	Forecast Class VBc < 0.2 mm
Actual class VBc ≥ 0.2 mm	83 (TP)	12 (FN)
Actual class VBc < 0.2 mm	3 (FP)	266 (TN)
Accuracy	0.959	
Precision	0.965	
Sensitivity	0.874	
F1	0.917	

**Table 8:** Quality measures of the classification diagnostic model based on the RF multidimensional prediction model (full set of SM_i_).

	Forecast Class VBc ≥ 0.2 mm	Forecast Class VBc < 0.2 mm
Actual class VBc ≥ 0.2 mm	88 (TP)	7 (FN)
Actual class VBc < 0.2 mm	3 (FP)	266 (TN)
Accuracy	0.973	
Precision	0.967	
Sensitivity	0.926	
F1	0.946	

**Table 9:** Results of preliminary tests of models on the training set.

	Accuracy	
Model	Full setof SM_i_	Reduced set of SM_i_
CART	0.881	0.881
RF	0.969	0.966
NNA	0.743	0.726
MLP	0.988	0.960

**Table 10:** Quality measures of the classification MLP diagnostic model based (full set of SM_i_).

	Forecast Class VBc ≥ 0.2 mm	Forecast Class VBc < 0.2 mm
Actual class VBc ≥ 0.2 mm	88 (TP)	7 (FN)
Actual class VBc < 0.2 mm	3 (FP)	266 (TN)
Accuracy	0.973	
Precision	0.967	
Sensitivity	0.926	
F1	0.946	

**Table 11:** Quality measures of the classification RF diagnostic model based (full set of SM_i_).

	Forecast Class VBc ≥ 0.2 mm	Forecast Class VBc < 0.2 mm
Actual class VBc ≥ 0.2 mm	89 (TP)	6 (FN)
Actual class VBc < 0.2 mm	4 (FP)	265 (TN)
Accuracy	0.973	
Precision	0.957	
Sensitivity	0.937	
F1	0.947	

**Table 12:** Comparison of the accuracy of different diagnostic models.

**Predictive Models**	**RMSE**	**σ_RMSE**	**R^2^**	
(1) One-dimensional (for Fy_amp, sqrt)	0.0491	0.0271	0.696	
(2) Multidimensional:				
• RF (full number of SM_i_)	0.0117	0.0009	0.9573	
• RF (reduced number of SM_i_)	0.0380	0.0009	0.949	
**Classification models**	**Accuracy**	**Precision**	**Sensitivity**	**F1**
(1) From regression models:				
• SR	0.865	0.735	0.758	0.746
• RF (full number of SM_i_)	0.959	0.965	0.874	0.917
• RF (reduced number of SM_i_)	0.973	0.967	0.926	0.946
(2) From raw data—full number of SM_i_:				
• MLP	0.973	0.967	0.926	0.946
(3) From raw data—reduced number of SM_i_:				
• RF	0.973	0.957	0.937	0.947

## Data Availability

Data will be made available on request.
